# Radiation-Induced Carotid Artery Stenosis in a Patient with Carcinoma of the Oral Floor

**DOI:** 10.1155/2013/379039

**Published:** 2013-06-01

**Authors:** Kahori Seto, Kenji Yamagata, Fumihiko Uchida, Toru Yanagawa, Kojiro Onizawa, Hiroki Bukawa

**Affiliations:** ^1^Department of Oral and Maxillofacial Surgery, Faculty of Medicine, University of Tsukuba, 1-1-1 Tennodai, Tsukuba City, Ibaraki 305-8575, Japan; ^2^Department of Oral and Maxillofacial Surgery, Mito Kyodo General Hospital, Ibaraki 310-0015, Japan

## Abstract

Radiation-induced carotid artery stenosis (RI-CS), a life-threatening condition, can occur after external radiation for head and neck cancer. We here describe a case of asymptomatic RI-CS in a 73-year-old patient treated with chemoradiotherapy and radical neck dissection for a basaloid squamous cell carcinoma of the oral floor. Stenosis of the left carotid artery, diagnosed as RI-CS, showed on an MRI performed 1.5 years after radiotherapy. Blood from the left side of the anterior cerebral artery and the middle anterior artery was flowing to the brain through the anterior and posterior communicating arteries, so no stent surgery or other treatment was necessary. The cancer has not recurred during approximately 5 years of followup after radiotherapy, and the patient has had no adverse effects from the RI-CS since it was diagnosed 3.5 years ago. This case emphasizes the necessity of early scrutiny for RI-CS in patients given radiotherapy for oral cancer.

## 1. Introduction

External beam radiotherapy is often necessary to treat cancers of the oral cavity, pharynx, larynx, or salivary gland, or lymphomas involving the cervical lymph nodes. Radiation-induced carotid artery stenosis (RI-CS), a life-threatening complication, is reported to occur in 30% to 50% of patients treated with external irradiation for head and neck cancer (HANC) [1]. Patients with RI-CS have an increased risk of stroke, and the risk increases with hypertension, diabetes, smoking, or obesity. Although the radiation port for treating HANC always includes the carotid arteries, the factors responsible for later effects of radiation on these large blood vessels have not been adequately defined [2–4].

The prevalence of RI-CS varies according to the primary cancer site. Although independent significant predictors reported for RI-CS include nasopharynx and larynx cancer, only 1 of 25 (4.0%) patients treated for tongue cancer developed RI-CS [5, 6]. No [7] large studies of RI-CS occurring after treatment for carcinomas of the tongue and the oral floor have been conducted. We here report a rare case of asymptomatic RI-CS that developed in a patient treated with radiotherapy for carcinoma of the oral floor.

## 2. Case Report

**Figure 1:**
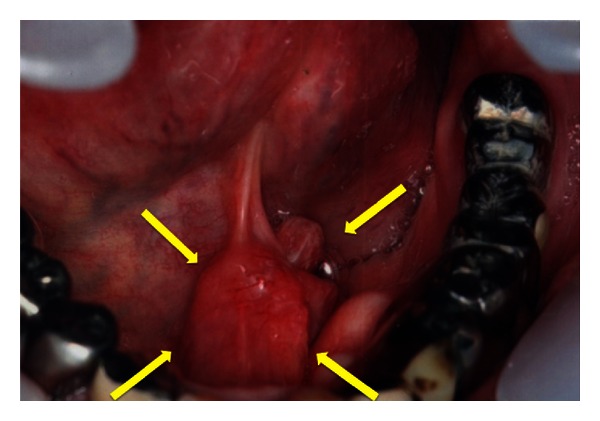
Examination showed a 22 × 15 mm mass with a granular surface in the middle of the mouth floor.

**Figure 2:**
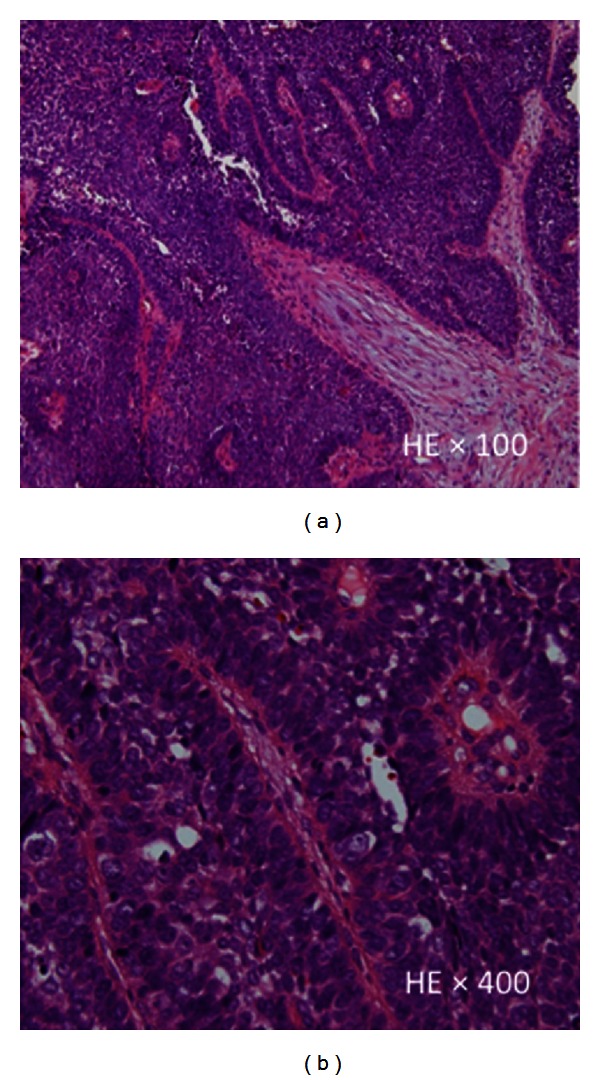
Lobulate or papillary alveolar forms were constructed by epithelial-like and basaloid tumor cells. These cells showed peripheral palisading, hyperchromatic nuclei with a high N/C ratio, and frequent mitosis (HE, ×100 and ×400).

A 73-year-old man was referred to the University of Tsukuba Hospital with a mass in the floor of his mouth. His medical history included hypertension and asthma. He had smoked 20 cigarettes a day for 30 years, until the age of 58. He denied any symptoms of cerebrovascular or hepatic disease. His face appeared symmetrical, and the submandibular lymph nodes on both sides were soft and soybean sized. Examination showed a 22 × 15 mm unmovable mass with a granular surface and a hard, elastic texture in the middle of the floor of the mouth (Figure 1). Histopathology led to a diagnosis of basaloid squamous cell carcinoma (BSCC) (Figure 2). With a diagnosis of carcinoma of the oral floor (T2N2bM0), the patient underwent concurrent chemoradiotherapy with a first course of 5-FU (600 mg/m^2^), TXT (50 mg/m^2^), and CDGP (60 mg/m^2^), and a second course of S-1 (100 mg/day) combined with radiotherapy (45 Gy to the neck and 69 Gy to the primary lesion). A biopsy did not find viable cancer cells remaining at the primary site. Since computerized tomography (CT) scans indicated possible metastases remaining in the cervical lymph nodes, we performed left radical and right supraomohyoid neck dissection. The patient's recovery period was uneventful, and the cancer did not recur during a 5-year followup period.

**Figure 3:**
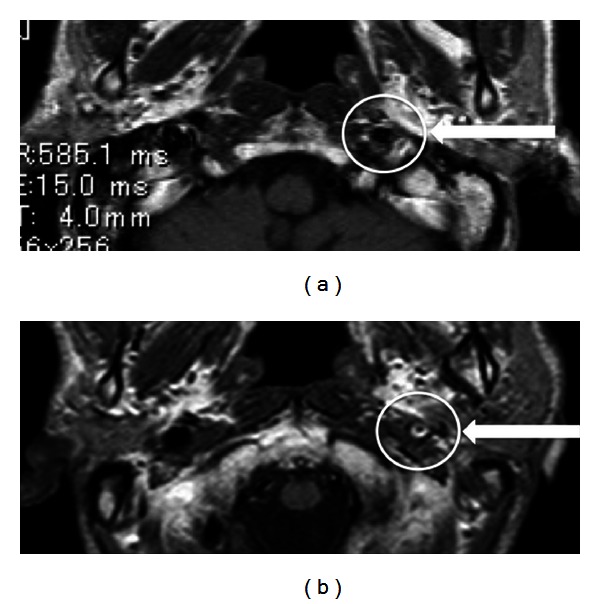
MR imaging (T2). (a) A flow void at the left carotid artery was observed immediately after radiotherapy. (b) No flow void was seen 1.5 years after radiotherapy, suggesting occlusion of the carotid artery.

**Figure 4:**
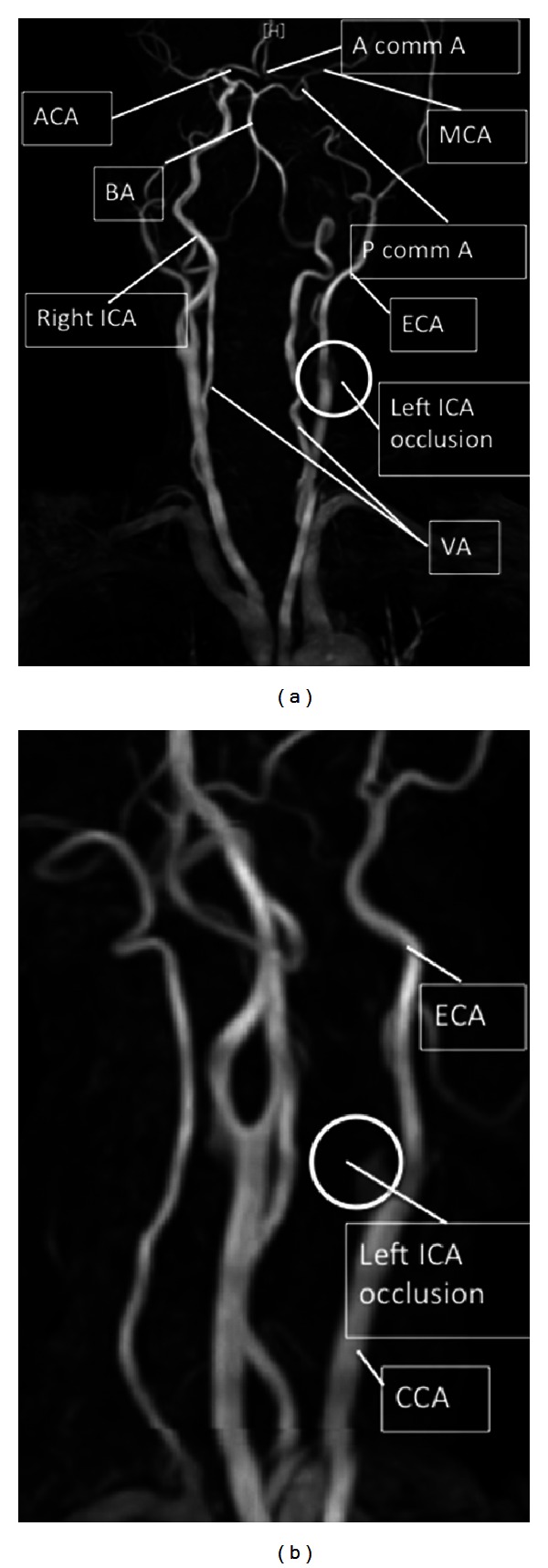
MRA showed an occluded carotid artery 1 cm from the origin (arrows). The anterior and middle cerebral arteries were being nourished through the anterior and posterior communicating arteries. A comm A: anterior communicating artery, ACA: anterior cerebral artery, P comm A: posterior communicating artery, BA: basilar artery, CCA: common carotid artery, ICA: internal carotid artery, ECA: external carotid artery, MCA: middle cerebral artery, and VA: vertebral artery.

A magnetic resonance imaging (MRI) conducted immediately after radiotherapy treatment confirmed the presence of flow void in the left carotid artery and no arterial occlusion (Figure 3). The flow void was obscure on an MRI performed 1.5 years after radiotherapy, suggesting severe RI-CS (Figure 3). The MRI showed chronic ischemia-related changes and several high-signal-intensity regions, including a spot in the cerebral white matter. Magnetic resonance angiography (MRA) showed an occlusion at the internal carotid artery 10 mm above the carotid bifurcation (Figure 4). Based on these scans and the history of chemoradiotherapy, our neurosurgery department diagnosed RI-CS. Carotid artery stenting (CAS) or carotid endarterectomy (CEA) was not performed because the patient was asymptomatic; the middle anterior artery and the left side of the anterior cerebral artery were fed by the posterior and anterior communicating arteries. In the 3.5 years since the RI-CS was discovered, the patient has not suffered any adverse events.

## 3. Discussion

As survival improves for patients with HANC, the long-term effect of cervical radiotherapy on the carotid vessels has become more apparent [7]. Case reports of atherosclerosis-like changes in carotid arteries and other large vessels after radiotherapy began to emerge in the 1970s [8, 9]. Cheng et al. found a significant correlation between carotid artery stenosis of 70% or more and an age of 60 years or more, a history of cerebrovascular symptoms, previous irradiation for nasopharyngeal or laryngeal carcinoma, or a postradiotherapy interval of 5 years or more [5].

An RI-CS diagnosis is suggested by Avitia's criteria: (1) a history of radiotherapy involving the neck, (2) no evidence of contralateral stenosis, and (3) a longer-than-usual stenotic segment [10]. Our patient had two of the three criteria. Mechanisms of RI-CS include (1) damage to the vasa vasorum, causing ischemic necrosis with subsequent fibrosis, (2) adventitial fibrosis with narrowing, and (3) acceleration of the atherosclerotic process [6]. Risk factors for RI-CS and stroke include hypertension, smoking, diabetes, dyslipidemia, obesity, and aging [11–13]. Our patient's medical history included the known risk factors of hypertension, an age greater than 70, and a history of 30 years of smoking 20 cigarettes per day. RI-CS developed more quickly in this patient after radiotherapy than in other reported cases.

A study of the incidence of RI-CS after radiotherapy for HANC found significant carotid stenosis in 30% of patients treated with radiotherapy involving the head and neck, but in only 5.6% of subjects not treated with radiotherapy [14]. Another study found significant RI-CS in 18%–40% of patients at 7.5 to 10 years after radiotherapy [7, 15]. The actual risk of stroke increased exponentially after 10 years, with strokes escalating to 14.0 per 1,000 patients per year after radiotherapy [16]. Our patient's asymptomatic RI-CS was discovered 1.5 years after radiotherapy, which is significantly earlier than in other reports. RI-CS was observed by coincidence in the course of CT or MRI scans conducted every 3 months to monitor cancer recurrence. This RI-CS developed quickly and, in the absence of frequent periodic exams, might have put the patient at risk of a stroke.

Therapeutic options for symptomatic stenotic lesions after radiotherapy include percutaneous transluminal angioplasty with or without stenting [17], CEA [18], and bypass surgery [15]. Early treatment is required in the case of a cardiac murmur, an ischemic attack, a cognitive drop, or a temporary visual disorder, such as the amaurosis of one eye. Our patient was asymptomatic. The left side of the anterior cerebral artery and the middle anterior artery were found to be fed by the anterior and posterior communicating arteries. Therefore, no treatment was required.

As HANC treatment evolves, the number of long-term survivors is increasing. While all radiation-treated patients have a high risk of developing RI-CS, those treated for HANC—especially those in higher-risk groups—should be followed more closely to permit early diagnosis. Physicians must be alert for RI-CS in patients treated with radiotherapy for oral cancer.
